# Supplementary material for: ZBTB18 inhibits SREBP-dependent lipid synthesis by halting CTBPs and LSD1 activity in glioblastoma
Source: Life Sci Alliance. 2022 Nov 22;6(1):e202201400. doi: 10.26508/lsa.202201400 (PMC9684030; doi:10.26508/lsa.202201400)
Supplement: Supplementary file 1 [file LSA-2022-01400_TableS1.docx]

**Table S1.** List of primers used for qRT-PCR.

| Primer name | Primer sequence |
| --- | --- |
| 18s_F | CGCCGCTAGAGGTGAAATTC |
| 18s_R | CTTTCGCTCTGGTCCGTCTT |
| ABCA1_F1 | ACCCACCCTATGAACAACATGA |
| ABCA1_R1 | GAGTCGGGTAACGGAAACAGG |
| LDLR_F1 | TCTGCAACATGGCTAGAGACT |
| LDLR_R1 | TCCAAGCATTCGTTGGTCCC |
| SREBF1_F1 | ACAGTGACTTCCCTGGCCTAT |
| SREBF1_R1 | GCATGGACGGGTACATCTTCAA |
| INSIG1_F2 | ATCCAGAGGAATGTCACTCTCTT |
| INSIG1_R2 | AGGGGTACAGTAGGCCAACAA |
| ACACA_F1 | ATGTCTGGCTTGCACCTAGTA |
| ACACA_R1 | CCCCAAAGCGAGTAACAAATTCT |
| FASN_F2 | CCGAGACACTCGTGGGCTA |
| FASN_R2 | CTTCAGCAGGACATTGATGCC |
| SCD_F2 | GCCCCTCTACTTGGAAGACGA |
| SCD_R2 | AAGTGATCCCATACAGGGCTC |
