# Supplementary material for: ZBTB18 inhibits SREBP-dependent lipid synthesis by halting CTBPs and LSD1 activity in glioblastoma
Source: Life Sci Alliance. 2022 Nov 22;6(1):e202201400. doi: 10.26508/lsa.202201400 (PMC9684030; doi:10.26508/lsa.202201400)
Supplement: Supplementary file 2 [file LSA-2022-01400_TableS2.docx]

**Table S2.** List of antibodies used for western blots.

| Antibody name | Company |
| --- | --- |
| mouse anti-FLAG | Sigma #F1804 |
| rabbit anti FLAG | (Cell Signaling #2368S) |
| rabbit anti-ZBTB18 | AbCam #ab118471 |
| mouse anti-CTBP2 | BD Biosciences #612044 |
| mouse anti-CTBP1 | BD Biosciences #612042 |
| rabbit anti-CTBP2 | Cell Signaling #13256S |
| rabbit anti-CTBP1 | Cell Signaling #8684 |
| rabbit anti-LSD1 | Millipore #17-10531 |
| mouse anti-LSD1 | Santa Cruz # sc-53875 |
| rabbit anti-ZNF217 | Thermo Fisher scientific #720352 |
| mouse anti alpha tubulin | Abcam #ab7291 |
