# Supplementary material for: ZBTB18 inhibits SREBP-dependent lipid synthesis by halting CTBPs and LSD1 activity in glioblastoma
Source: Life Sci Alliance. 2022 Nov 22;6(1):e202201400. doi: 10.26508/lsa.202201400 (PMC9684030; doi:10.26508/lsa.202201400)
Supplement: Supplementary file 3 [file LSA-2022-01400_TableS3.docx]

**Table S3.** List of primers used for cloning and site-directed mutagenesis.

| Primer name | Primer sequence |
| --- | --- |
| ZNF238_LDLmut_f | CTGAAAGGCTGGACTTGACAGACGAGGCCGACACACAGTCAACATCTGCCGAAT |
| ZNF238_LDLmut_r | ATTCGGCAGATGTTGACTGTGTGTCGGCCTCGTCTGTCAAGTCCAGCCTTTCAG |
| BstXI-flag-hZBTB18-sense | TGGCCACAACCATGGACTACAAGGACGACGATGACAAGTGTCCTAAAGGTTATGAAGACAG |
| PmeI-HA-hZBTB18-antisense | GCCTTGGTTTAAACTTAAGCGTAATCTGGAACATCGTATGGGTATTTCCAAAGTTCTTGAGAGCTA |
