# Supplementary material for: ZBTB18 inhibits SREBP-dependent lipid synthesis by halting CTBPs and LSD1 activity in glioblastoma
Source: Life Sci Alliance. 2022 Nov 22;6(1):e202201400. doi: 10.26508/lsa.202201400 (PMC9684030; doi:10.26508/lsa.202201400)
Supplement: Supplementary file 4 [file LSA-2022-01400_TableS4.docx]

**Table S4.** List of antibodies used for co-IP.

| Antibody name | Company |
| --- | --- |
| rabbit anti-CTBP2 | Cell Signaling #13256S |
| mouse anti-CTBP | Santa Cruz #sc-17759 |
| mouse anti-LSD1 | Santa Cruz # sc-53875 |
| mouse anti-FLAG | Sigma # F1804 |
| rabbit anti-ZBTB18 | AbCam #ab118471 |
| rabbit anti-ZBTB18 | Proteintech #12714-1-AP |
| normal mouse IgG | Santa Cruz, #sc-2025 |
| normal rabbit IgG | Santa Cruz, #sc-2027 |
