# Supplementary material for: ZBTB18 inhibits SREBP-dependent lipid synthesis by halting CTBPs and LSD1 activity in glioblastoma
Source: Life Sci Alliance. 2022 Nov 22;6(1):e202201400. doi: 10.26508/lsa.202201400 (PMC9684030; doi:10.26508/lsa.202201400)
Supplement: Supplementary file 5 [file LSA-2022-01400_TableS5.docx]

**Table S5.** List of antibodies used for ChIP.

| Antibody name | Company |
| --- | --- |
| rabbit anti-CTBP2 | Active Motif #61262 |
| mouse anti-FLAG | Sigma #F1804 |
| rabbit anti-LSD1 | Merck Millipore #17-10531 |
| rabbit anti-ZNF217 | Thermo Fisher Scientific #720352 |
| rabbit anti-H3K4me3 | Diagenode #C15410003 |
| rabbit anti-H3K4me2 | Cell Signaling #9725 |
| rabbit anti-H3K9me2 | Cell Signaling #9753 |
| mouse anti-H3K9me2 | Abcam #ab1220 |
