# Supplementary material for: ZBTB18 inhibits SREBP-dependent lipid synthesis by halting CTBPs and LSD1 activity in glioblastoma
Source: Life Sci Alliance. 2022 Nov 22;6(1):e202201400. doi: 10.26508/lsa.202201400 (PMC9684030; doi:10.26508/lsa.202201400)
Supplement: Supplementary file 6 [file LSA-2022-01400_TableS6.docx]

**Table S6.** List of primers used for quantitative ChIP.

| Primer name | Primer sequence |
| --- | --- |
| LDLR_ChIP_TSS_Fw1 | ATAGAAAGTGGCGGAAGTTCC |
| LDLR_ChIP_TSS_Rv1 | ATAGAAAGTGGCGGAAGTTCC |
| FASN_ChIP_Fw | GGACGAAATGGGGATAGCCTA |
| FASN_ChIP_Rv | CTGTGGTGTGTGGGTTGGTAT |
| GSK3A_Fw | GGAAAGGCATCTGTCGGGG |
| GSK3A_Rv | GAGTGGCTACGACTGTGGTC |
| INSIG1_ChIP_fw | CCTTCCTCGCTCTTTGTCTCT |
| INSIG1_ChIP_rv | GTTGATCATCTCCCCAACCTT |
| SCAP_ChIP_fw | TGAGGTCATAAACCCACTCAGA |
| SCAP_ChIP_rv | TAGAACCTGCTTTGGTGCTGT |
| LSS_ChIP_fw | TGCGTGGTTTAGAGATGAAGG |
| LSS_ChIP_rv | CTGGACACCGTAAGTTGCTTC |
| ACACA_ChIP_fw | AGTTCCCTCAGCCTCAATTTC |
| ACACA_ChIP_rv | CTGACTTTTGATCCGACCAGT |
| CYP51A1_ChIP_fw | TGAGTCTTTGGCTTTCGTACC |
| CYP51A1_ChIP_rv | CAAGACAATCCCACCAAGATG |
| SQLE-Z-fw | TGCGACGGTTACTCTGGTTAC |
| SQLE-Z-rv | CCAGGGTACCTCCCTCAGAT |
| SREBF1-fw1 | CCCTCTGTAATGGTGTGCCTA |
| SREBF1-rv1 | AAGCGCTCAGCAAGTAAACTG |
